# Physiologically Based Pharmacokinetics Modeling in the Neonatal Population—Current Advances, Challenges, and Opportunities

**DOI:** 10.3390/pharmaceutics15112579

**Published:** 2023-11-03

**Authors:** Jean Dinh, Trevor N. Johnson, Manuela Grimstein, Tamorah Lewis

**Affiliations:** 1 Certara UK Limited, Sheffield S1 2BJ, UK; jean.dinh@certara.com (J.D.); trevor.johnson@certara.com (T.N.J.); 2 Office of Clinical Pharmacology, Center for Drug Evaluation and Research, U.S. Food and Drug Administration, Silver Spring, MD 20903, USA; 3 Pediatric Clinical Pharmacology & Toxicology, The Hospital for Sick Children, Toronto, ON M5G 1X8, Canada

**Keywords:** neonates, PBPK modeling, pharmacokinetics, ontogeny

## Abstract

Physiologically based pharmacokinetic (PBPK) modeling is an approach to predicting drug pharmacokinetics, using knowledge of the human physiology involved and drug physiochemical properties. This approach is useful when predicting drug pharmacokinetics in under-studied populations, such as pediatrics. PBPK modeling is a particularly important tool for dose optimization for the neonatal population, given that clinical trials rarely include this patient population. However, important knowledge gaps exist for neonates, resulting in uncertainty with the model predictions. This review aims to outline the sources of variability that should be considered with developing a neonatal PBPK model, the data that are currently available for the neonatal ontogeny, and lastly to highlight the data gaps where further research would be needed.

## 1. Introduction

The pharmacology modeling tools available for research, and translational to clinical care, have expanded greatly in the past thirty years. Physiologically based pharmacokinetic (PBPK) modeling is a scientific approach allowing the incorporation of complex physiologies in drug absorption, distribution, metabolism, and excretion to predict drug pharmacokinetics. This modern modeling technique has been adopted by multiple stakeholder groups, including academic researchers, industry, and regulatory agencies [1]. PBPK modelling has been used for a variety of purposes, including, but not limited to, in silico prediction of exposure to choose doses for clinical trials, understanding the influence on drug exposure of impaired organ function, predicting drug–drug interactions, and predicting fetal exposure to maternal medications during pregnancy.

PBPK modeling is an important tool in understanding how to optimize dosing in patient subpopulations who can either not be enrolled in clinical trials or for which there is limited information from drug-development efforts. Examples of such subpopulations include pregnant and lactating women, fetuses, neonates and infants, and other pediatric groups [2,3,4]. A major cornerstone of PBPK modeling is the incorporation of unique patient physiology, and it is thus a powerful tool for anticipating how drug PK could differ in these populations compared to populations more extensively studied [5,6,7]. Additionally, this tool can be used for in silico predictions aimed at the rational choosing of “first in population” doses for these patients, when traditional allometric scaling may be less accurate. This review will focus specifically on the potential for PBPK modeling to transform pharmacology research and clinical implementation in neonates.

**Figure 1:**
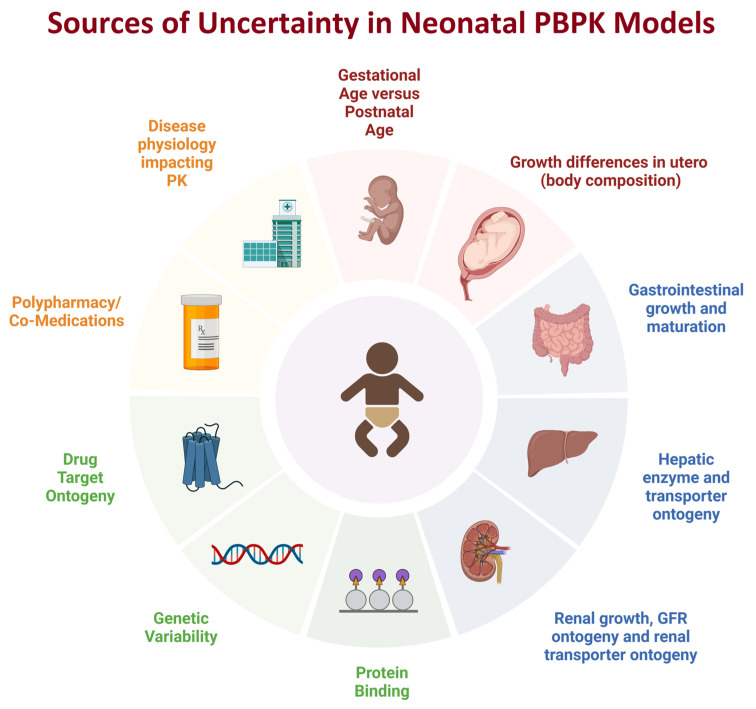
Sources of uncertainty when developing neonatal PBPK models (created with biorender.com). The source of uncertainty may be related to variability in the neonatal population for an age category (e.g., the developmental differences between preterm and term child whose postnatal age is one month). Some sources of uncertainty are due to lack of data, e.g., drug target ontogeny whereas other sources of uncertainty are due to the complexity of the question, such as the impact of co-medications and potential DDIs in a neonate, or the impact of disease in neonates impacting drug PK and PD.

Neonates are typically defined as newborns at less than 1 month of life, but there is actually a diverse patient population captured within this umbrella designation, which is illustrated in Figure 1. For example, a neonate can range in gestational age (GA) at birth from 22 weeks (limit of viability) to 41 weeks (post-term), and in birth weight from 400 g to 4500 g. Neonates can have a wide range of developmentally programmed organ function (gastrointestinal, renal, and hepatic) that can vary widely between GA and postnatal ages (PNA) [8,9]. Since each of these neonatal subpopulations are small in number, large studies are not feasible. Thus, PBPK models have great potential to use unique physiology and drug characteristics to augment our understanding of the dose-exposure response. Additionally, PBPK models can help us understand tissue-specific drug exposure and unique neonate-specific drug toxicities (i.e., neurodevelopmental sequelae of drug exposure in the developing brain) [10]. Of note, because preterm infants are a very unique and vulnerable neonatal subpopulation, many PBPK software groups have incorporated ontogeny profiles specifically for this population. 

## 2. General Principles of Developing PBPK Models in Neonates

Currently, there are several software programs available to assist researchers in developing PBPK models in the neonatal populations. A non-exhaustive list of some commercially available PBPK Modeling Software includes Simcyp^®^ (Certara UK Limited, Sheffield, UK), Gastroplus^®^ (SimulationsPlus, Cognigen, Lancaster, CA, USA), PK-Sim^®^ (Bayer Technology Services, Leverkusen, Germany), etc. The architecture of each program is not the focus of this paper; however, it is worthwhile to describe the general approach and data that PBPK software use to simulate drug PK in special populations [11,12,13,14,15,16,17,18]. Readers are encouraged to review the publications for each software for further details prior to building any models. Ultimately, each PBPK software package simulates PK in special populations using two categories of data: systems data (i.e., patient population data) and compound data (i.e., drug and drug-formulation data). The systems data describes the physiological parameters of a population that impact PK. Important physiological parameters include plasma protein binding, organ volumes, organ function (e.g., kidney glomerular filtration rate), blood flow, tissue composition, enzyme and transporter abundance, etc. For neonates, the incorporation of an ontogeny (developmental profile) to these physiologies is critical to anticipating drug PK. Therefore, if an ontogeny is not built into the systems data or if the ontogeny data are sparse, unreliable, or lack validation, this is an important limitation to the function of PBPK model performance in neonates. As such, robust data describing neonatal differences in physiology are key to developing high-quality PBPK models for neonates. Table 1 describes several of the key parameters and processes incorporated in PBPK models, and the strength of information available for neonates.

The second category of data is compound data, which comprises drug-specific data relating to the drug’s physical and chemical properties, the solubility/permeability of the compound, tissue distribution, metabolism, and excretion of the compound. While these data are generally less impacted by differences between adult and pediatric populations, it should be noted that many key in vitro experiments that predict the metabolism and excretion of compounds are performed using adult tissues. The underlying assumption of many PBPK models is that the function of a protein or enzyme does not differ between adults and neonates. Thus, this could be considered a major assumption necessitated by a current “data gap” to consider when building neonatal PBPK models.

While the general science of PBPK modeling has matured considerably, there is considerable room for growth in the neonatal space due to (1) remaining data gaps for optimal model specification and (2) the low number of pharmacometricians and pharmacologists focused on this specific patient population. Ultimately, one of the major purposes of developing a PBPK model for neonates is to help determine safe and effective doses of medication for these vulnerable patients, in the absence of the ability to conduct large clinical trials in this population. For example, if we knew the target drug exposure for a new antibiotic from adult clinical trials, a PBPK model could incorporate neonatal physiology to predict the dose and dose frequency required to obtain this target exposure in a neonate. In a second example, if we know a drug concentration associated with toxicity in neonates, PBPK models can be used to test, in silico, different dosing strategies to avoid toxic drug exposures. In this review, we also aim to highlight the key areas of data “need” from a research, clinical, and regulatory perspective. We believe that identifying and systematically addressing these needs as a scientific community can accelerate the development of robust PBPK models for clinical decision making.

## 3. Advances and Unique Challenges in Developing PBPK Models in Neonates

Several recent publications have highlighted the sparse use of PBPK modeling in both term and preterm neonatal populations [131,132,133]; many pediatric PBPK studies only go down to the infant population and no younger. The reasons for this lack of research include relatively low amounts of published clinical PK data in neonates (needed to validate the PBPK models) and general uncertainty regarding this approach in the very young due to lack of good-quality systems data.

Nevertheless, important developments within pediatric PBPK models, particularly in reference to younger age groups, have been made. One highly impactful development is the introduction of time-based changing physiology [12], whereby subjects can be redefined over time, and thereby changes due to growth can be incorporated into the PBPK model. Before this development, a research patient had to stay static over time in the model, despite medication dosing over weeks or months. This approach works in adults who do not have rapidly changing body size or organ maturation. But time-based changing physiology is particularly important in neonates who are rapidly growing and maturing in a short time frame. An example of incorporation of time-based changing physiology can be seen when performing PK simulations of sildenafil, a drug that is dosed in neonates with pulmonary hypertension over multiple weeks [12,134]. Mukherjee et al. reported that sildenafil clearance increased substantially over the first 7 days of life, which is likely a result of increased CYP3A4 activity during this period [134]. A static model would underestimate clearance, and thus the recommended dose from this model could potentially be subtherapeutic. On the other hand, a PBPK model that incorporates the ontogeny of CYP3A4 in the first days of life is better able to capture clearance, and this insight could lead to better dose adjustments during the entirety of sildenafil treatment for a neonate [12].

Another important development is the ability to account for GA as well as PNA [13,135] in neonatal PBPK models. This new developmental allows for simulations in preterm infants, which allows for capturing PK in a neonatal subpopulation that is developmentally less mature. Additionally, accounting for GA and PNA will also help to further verify data in term neonatal models, once the preterm born infants mature towards term physiology.

Recent publications have specifically compiled physiological data for building PBPK models in fetus, preterm, and term neonates [11,13,135]. Rather than describe the individual systems data in detail, the current scientific data regarding the key PBPK parameters in the preterm and term neonatal populations, the amount of data available for each parameter, and important knowledge gaps is summarized in Table 1. The following review sections will be used to illustrate how the systems data in neonatal PBPK models can be improved.

### 3.1. Demographic Data

Age, weight, and length are the demographic variables needed to build PBPK models. Many growth charts exist for different populations covering both preterm and term neonates [136,137]. Most current neonatal PBPK models use weight equations based on PNA, but allowing for postnatal dip [17], or postmenstrual age (PMA) [7]. Some longitudinal data is available for parameters such as height and weight, and this has been used to develop equations describing these parameters from preterm (25 weeks GA) to 4 years of age [138]. Using this approach, ‘catch up’ growth in preterm infants can be described. Future development will include integrating these types of growth models into neonatal PBPK such that height and weight are fully described in relation to both GA and PNA.

### 3.2. Organ Size, Blood Flow, and Composition

This information is required for the PBPK model to allow prediction of the drug distribution to different tissues. In general, there is a good amount of information on organ size in term and preterm neonates, and underlying algorithms describing changes can be linked to relevant covariates such as age, height, and weight but also other parameters such as fat free mass (e.g., muscle volume). Taking the liver as an example, a meta-analysis of age-related changes to liver volume included 576 term neonates; the volume was described in relation to body surface area [17]. For the preterm population, liver volume has been defined in relation to body weight [13]. Changes to cardiac output with age are well-defined in preterm and term neonates [57,58]; however, individual tissue blood flow is less well-defined, especially for preterm. There is limited data on hepatic blood flow changes in neonates [139], and this is important not only for volume-of-distribution prediction but also prediction of drug elimination. Drugs that have a high hepatic extraction are more dependent on hepatic blood flow for elimination. However, there is evidence that the extraction ratio may change with age, exemplified by morphine, which was shown to be more influenced by UDP-glucuronosyltransferase (UGT) 2B7 abundance rather than hepatic blood flow in neonates [140]. Whilst the extracellular water percentage of body weight decreases with age in neonates, there is a corresponding increase in intracellular water [141]. Data on individual tissue composition in terms of percentage water and fat in tissues in term neonates is sparse [36,37].

In general terms, the major problem with systems information available in neonates is that often what is reported is population mean data obtained from one or more cross-sectional studies where a wide age range was studied. In this situation, the true developmental patterns can be hidden. Additionally, this also leads to the overprediction of variability in future PK studies. There is a pressing need for longitudinal studies conducted in the same individuals over time; in this way, the changes in systems parameters, and the relation to both GA and PNA, can be better defined; although, at the same time, it is acknowledged that these studies are more difficult to perform. In the light of this, there has been a call for construction of centralized databases on physiological parameters based on longitudinal real-world data [131].

### 3.3. Ontogeny of Oral Drug Absorption

There is some evidence for changes in the rate and extent of oral absorption, particularly in preterm and term neonates [142,143], and there is ongoing research in this area [144]. One area of current interest is predicting the effects of age on the oral absorption of drugs in children, and existing PBPK models of absorption have been extended to include information on age-related changes in these system parameters [14,145]. However, extending these models to especially preterm neonates is challenging due to conflicting data regarding key system parameters and potential confounders that may influence the data, such as how fasted/fed states are defined in neonates. 

A key example is how gastric emptying changes with age. A model-based meta-analysis [29], including preterm and term neonates, has shown that mean gastric residence time does not change with age but is affected by meal type. This study illustrates potential confounding effects, because subjects on liquid feeds had faster gastric emptying compared to those on solid feeds but, by definition, neonates receive liquid feeds. This study was also significant in that it went against the preconceived ideas regarding gastric-emptying patterns in neonates and a few review publications suggesting that gastric emptying was slower in neonates [141,146]. 

A separate meta-analysis on mean small intestinal transit time [30] also showed no change with age, but only included one study on preterm and term neonates [147]. There is some evidence of changes in intestinal permeability in preterm infants [148] but more research is needed in this area. There is data on the ontogeny of intestinal cytochromes P450 (CYP) 3A4 enzyme and some intestinal transporters (see Table 1). The ontogeny of intestinal CYP3A4 shows an increasing pattern from fetus to neonate and then early infant [16], and this is reflected in the increased bioavailability of oral midazolam seen in the preterm [149]. More basic and clinical research is needed to expand the absorption models down into the preterm populations with confidence.

### 3.4. Ontogeny of Hepatic and Renal Drug Elimination Pathways

Most ontogeny data for hepatic elimination pathways such as CYPs, UGTs, and transporters is limited in the neonatal populations, and there are certainly few studies with relatively rich data in the preterm or 0 to 1 month age groups. For example, in the study by Mooij et al. [31], relatively rich mRNA data are presented for ontogeny of hepatic P-gp in fetuses (n = 9) and neonates of 0 to 1 month (n = 16), whereas in the proteomic study by Prasad et al. [118] there are only four subjects in the neonatal group. There is generally much less data in the preterm populations, and this limits the extension of the models to lower GA. Since access to fetal and neonatal liver tissue has been an issue, the establishment of more extensive liver banks would help address this knowledge gap. Some ontogeny studies do not report data in neonates [150], or because of low subject numbers use non-standard age groups, e.g., (0 to 3 months) to report values, meaning that actual neonatal data is lost [151]. Most ontogeny studies on CYPs, UGT, other enzymes, and transporters are from in vitro data (mRNA, proteins abundance, probe drug activity) which may not reflect in vivo activity with age. In the pediatric age range, two studies have presented in vivo-derived ontogeny data based on the deconvolution of data from clinical studies [75,76]. Ideally, this sort of approach may be applied to the neonatal populations but is more difficult, as clinical studies involving specific probe drugs such as midazolam in these ages tend to be used in subjects with serious medical conditions where disease effects can be significant. 

A key element of describing drug metabolism within a neonatal PBPK framework is the incorporation of both enzyme abundances (with applied ontogeny) and genetically predicted phenotype information (pharmacogenetics). Phenotype information in terms of ultrarapid, extensive, intermediate, and poor metabolizers is generally fixed within specific ethnic populations within PBPK modeling software tools (e.g., the Japanese population). Because both enzyme ontogeny and genetically predicted phenotype are described in the models, they can be used to replicate studies investigating the interplay of these two factors in neonates, infants, and children. The models can also aid in identifying the age at which a genetic phenotype can be identified or becomes relevant based on sufficient enzyme expression. Separating out the effects of genotype/phenotype from ontogeny, in terms of the determination of the latter as systems parameters in pediatric PBPK models, is particularly important (and difficult) in terms of the accuracy of pediatric PBPK modeling, and particularly in the early neonatal ages. For CYP2B6, an in vitro study [81] that included 24 fetal and 141 pediatric liver samples found there was a significant association with age but not genotype. When considered cumulatively, other in vitro and in vivo studies on CYP2D6 [87,152] may imply that the CYP2D6 genotype, and not ontogeny, is the primary source of variability in this enzyme in children [153]. Performing ontogeny studies in fixed phenotype subjects (e.g., only extensive metabolizers) can help get around these issues, but this makes subject recruitment and obtaining a sufficient sample size more difficult.

There are a number of drug-metabolizing enzymes such as CYP3A7 [90] or flavin-containing monooxygenase (FMO) 1 [96], with known fetal forms of the enzymes, and possibly some transporters, e.g., ABCG2 [119], where there is over-expression in preterm neonates and neonates compared to adults. There has been a lot of interest in the role of CYP3A7 both in the metabolism of drugs in neonates [154] and also its possible role in drug–drug interaction prediction [155]. Recombinant CYP3A7 is available commercially and can be used to screen drugs used in the neonatal populations in terms of in vitro metabolism. Studies using recombinant enzymes have shown low activity of CYP3A7 compared to CYP3A4 in the metabolism of midazolam [156], sildenafil [157], and oxycodone [158]. However, one of the problems with this approach has been the lack of scaling factors regarding the link between recombinant human enzyme activity compared to CYP3A7 within fetal and neonatal liver microsomes. A recent study using human fetal microsomes [158] reported an intersystem extrapolation factor for this enzyme which can be incorporated into PBPK models and will allow further evaluation of this enzyme’s role in neonatal drug elimination.

### 3.5. Defining Age and Maturation in a Neonatal Population

For the preterm population, added complexity arises due to the influence of both GA and PNA on systems parameters, as already indicated above. Few or no studies currently exist on the ontogeny of drug-metabolizing enzymes based on GA and PNA. However, a recent study [121] has described the ontogeny of the glomerular filtration rate (GFR) based on this approach. Using inulin data, the study demonstrates that GRF increases as a function of both GA and PNA, and that birth has an impact on GFR value. Neonates with the same postmenstrual age who have been born longer have higher GFR values compared with less premature but younger neonates. Incorporating this interplay between GA and PNA on system parameters into preterm PBPK models is an area for future research. One of the key parameters to improve the accuracy of ontogeny descriptors within neonatal PBPK models is the fraction of adult expression/activity at term birth and at the various GA for preterm birth. More data in this area will help in improving both preterm and term PBPK models.

One criticism of current models is the dependence on data derived from relatively healthy subjects rather than from neonatal subjects with diseases [131]. However, logically general PBPK models for both preterm and term neonates can be developed based on ‘healthy’ individuals and then extended to more specific subpopulations based on disease or genetic ancestry, as is done for adult populations [159,160]. How drug disposition and clearance differ between relatively healthy preterm neonates and those with multiple comorbid diagnoses is an important area for future development. 

Another area for improvement is how variability is defined in neonatal PBPK models. For certain parameters such as height and weight, there is sufficient data to define variability with both GA and PNA. However, for other parameters, such as CYP expression in the liver, variability is unknown, and the variability is assumed in the modeling process to be the same as the general adult population. This similar degree of variability to adults may not be the case, and variability for certain enzymes (e.g., CYP1A2) may be lower due to less exposure to xenobiotics.

### 3.6. Biologics

There is increasing therapeutic use of large molecules such as monoclonal antibodies in pediatrics, including some that are used in neonates (e.g., palivizumab) [161]. The existing PBPK-modeling approach for these drugs has been extended to pediatrics [162], including preterm neonates [163]. Further information is needed on the ontogeny of some of the key parameters that influence the PK of these drugs, including the neonatal Fc receptor (FcRn) and specific target ontogeny, the latter as mediators of target-mediated drug disposition. 

### 3.7. Ontogeny of Drug Response

While this review focuses on the knowledge gaps surrounding biological processes involved in drug pharmacokinetics, it should be acknowledged that there is an even greater paucity of information describing differences in drug response in the neonatal population. For example, we currently do not have a thorough understanding of the ontogeny of physiological systems and potentially associated drug target receptors. Another area where we lack knowledge is whether drug receptors in infants have differing affinities to small molecules compared to adult receptors. This lack of knowledge also prohibits our ability to anticipate off-target binding and potential adverse drug reactions. An example where the developmental patterns of a physiological system could have impact on PD is the neuronal GABA and glutamate systems in preterm children. Here, there have been observed concentration differences in GABA and glutamate between preterm babies and older children [164,165]. Maturation differences in baseline neurotransmitter levels may lead to a different drug response and side effects in drugs targeting the CNS in neonates. Indeed, this is an area of research that is much needed to accurately determine safe and effective dosing for the neonatal population.

## 4. PBPK and Regulatory Application in Neonates

Regulatory agencies, such as the U.S. Food and Drug Administration (FDA) and the European Medicines Agency (EMA), have implemented guidelines and projects to encourage and facilitate the development of pediatric drug products, including those specifically targeting neonates [166,167]. These initiatives aim to promote research in neonates, facilitate the availability of safe and effective drug therapies for this vulnerable population, and improve therapeutic outcomes in neonatal pharmacotherapy. Quantitative modeling approaches, such as PBPK and population PK, are highlighted as a critical component, along with adult clinical data and prior knowledge, to help inform neonatal drug development by optimizing the design and dose selection of neonatal studies [167]. It is noted, however, that relevant maturation data with respect to ADME characteristics would preferably be available for the development of a robust and accurate model before applying it for the intended purpose [167]

In the context of regulatory application, PBPK modeling in neonates is particularly challenging. There are still knowledge gaps related to the physiology and maturation/ontogeny in neonates which can affect drug ADME processes, and the intrinsic variability expected in this population (based on the age, body weight, and developmental stage). Qualification of model performance is an integral part of model development, aiming to determine if physiological changes have been adequately captured by the population model. Adequate model qualification informs the confidence in modeling predictions and is related to the intended purpose and regulatory impact of the modeling analysis [168]. However, qualification of a neonatal PBPK model may be hindered in most cases by the limited availability of relevant clinical data. The reader is referred to the FDA and EMA guidelines with regards to the general development and qualification of PBPK models [169,170].

Acknowledging model uncertainties, and evaluating their impact on model performance, is essential to instill model confidence in the neonatal population. For instance, fraction unbound in plasma (fup) has been identified as a key parameter driving pediatric PBPK model outputs [18]. For neonates, different ontogeny models had different a prediction accuracy for fup, especially for drugs highly bound to alpha-acid glycoprotein [171]. Notably, the maturation of absorption processes is not fully characterized. For example, the assertion that gastric emptying is slower in neonates than in older children and adults has been challenged [29]. Recognizing knowledge gaps allows the exploration of hypotheses in a neonatal PBPK analysis and informs future research.

By highlighting two examples of recent FDA drug approvals for pediatric indications in the following paragraphs, we illustrate the potential of PBPK modeling as a tool for selecting an initial neonatal dose and facilitating drug development in neonates.

**Figure 2:**
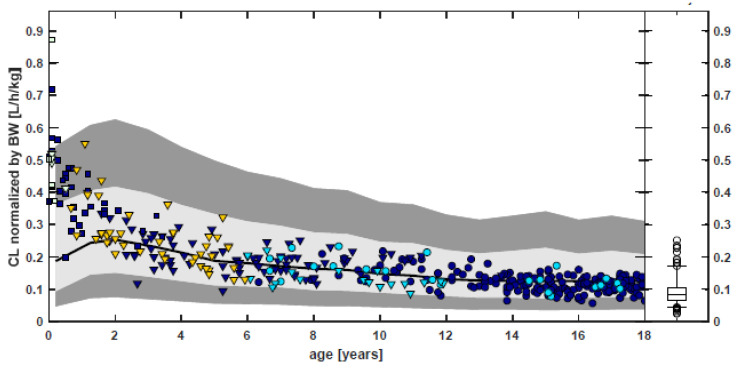
Comparison of PBPK predictions and population PK analysis estimates of rivaroxaban clearance normalized by body weight (bottom) as function of age. Symbols represent individual estimates using the population PK model for children. Black line: geomean of PBPK predictions of CL for a virtual pediatric population. Gray-shaded areas: 5th–95th percentile for a virtual pediatric PBPK population and enlarged expected range (0.5 × 5th–1.5 × 95th percentile). Box whisker plot: population PK results of the adult VTE (20 mg QD). [Source: Reference [172]: US Food Drug Administration, Office of Clinical Pharmacology Review—Xarelto. 2021.].

Following the “learn and confirm” paradigm, PBPK and population PK analyses were applied throughout the pediatric program of rivaroxaban, with a body weight-based dosing adjustment derived by PBPK modeling [172,173]. The pediatric dosing strategy was designed to achieve rivaroxaban exposure in pediatrics from birth to adolescence, that was similar to adult patients with deep-vein thrombosis receiving 20 mg once daily. Children were enrolled following an age-staggered, stepwise approach, starting with adolescents to term neonates. The results of the phase 1 studies in young pediatric cohorts (>6 months of age, body weight < 20 kg) showed lower exposure to rivaroxaban (based on the values of the area under the plasma concentration time curve over a 24 h dosing interval (AUC0-24h) and pre-dose trough concentration (Ctrough) at steady state) than initially predicted by the PBPK model, indicating a need for higher doses to achieve the target adult levels. No rationale in terms of physiological changes or maturation of processes affecting the ADME of rivaroxaban could be identified by the Sponsor to support adjustment of the pediatric PBPK model. Given the model limitations, for the neonatal study, intermediate doses (in mg/kg body weight) between those informed by the PBPK model and those already tested in children aged > 6 months in prior studies were selected. In neonates and infants (weighing ≤ 12 kg), the observed trough concentration values were also consistently at the lower end of the PBPK predicted range. Overall, for children aged <2 years, the predicted clearance was, on average, lower than population PK estimates. For children aged >2 years, the PBPK predictions of rivaroxaban clearance as a function of age were consistent with population PK estimates, including the predicted vs. observed range of interindividual variability. Figure 2 shows the PBPK predications compared to observed estimates of rivaroxaban clearance in children, including neonates. Therefore, clinical PK data and population PK analysis in children aged < 2 years informed dosing for the pediatric phase 3 study. For neonates and infants, a three-times-daily dosing regimen was required to increase the trough concentrations, targeting the adult exposure range [172].

In a second case example, PBPK analysis was used to support remdesivir dosing in pediatric trials, and dosing in the emergency use authorization (EUA) and pediatric compassionate use programs for treatment of COVID-19-infected patients as young as full-term neonates. Following the qualification of the PBPK model of remdesivir and plasma metabolites GS-704277 and GS-441524 with available PK data in adults, the model was applied to predict pediatric dosing using a virtual pediatric population aged 0 days to < 18 years. For children < 12 years of age and weighing > 3.5 kg, PBPK modeling informed the weight-based dosing regimen (a single loading dose of 5 mg/kg followed by 2.5 mg/kg/day i.v.) needed to achieve the target adult steady-state exposure range of remdesivir and plasma metabolites [174]. However, the FDA concluded that qualification of the PBPK model would require pediatric PK data, which were not available at the time of the original New Drug Application (NDA). Thus, the initial indication for remdesivir did not include children aged <12 years or weighing <40 kg. The FDA concluded that a dedicated pediatric trial to evaluate the safety, efficacy, and PK would be essential to provide support for an indication in this age group [175]. In the interim, access to remdesivir with dosing based on the PBPK model-selected remdesivir dose regimen for pediatric patients weighing > 3.5 to <40 kg was continued through EUA [176]. Subsequently, results from the pediatric study (GS-US-540-5823, clinicaltrials.gov identifier NCT04431453) were used to support expanding the indication to encompass pediatric patients 28 days and older and weighing at least 3 kg [177,178].

Noteworthily, providing the access of remdesivir to neonates/infants and children, through the EUA, demonstrated that the PBPK modeling approach can be used to improve pediatric treatment in urgent and unmet medical need situations [179].

Details about the PBPK model for remdesivir and rivaroxaban, including model development and validation, the scaling of the adult model to pediatrics, and its limitations are presented elsewhere [172,173,174,180].

## 5. Conclusions

For ethical reasons, healthy neonatal children are rarely enrolled in clinical trials where there is no prospect of benefit to the child. Since study populations are limited to those with diseases (often with co-morbid conditions), designing and completing traditional Phase 1-3 RCTs is challenging. For many old and new drugs used in neonates, data are not available to confirm optimal dosing guidelines, and when pharmacology studies are published, there is a delay in clinical implementation. Unfortunately, this creates a problematic scenario for clinicians and neonatologists who are tasked to determine a safe and effective dose of medication for a critically ill young patient. Modern pharmacometrics tools can help fill these knowledge gaps, and the development of comprehensive and robust neonatal PBPK models is absolutely needed. Indeed, the use of PBPK models represents one of the most viable strategies to anticipate drug exposure and response in the youngest children. The authors hope that this comprehensive discussion of research needs, particularly in transporter ontogeny and oral absorption ontogeny, will spur the scientific community to focus on this patient population for PBPK model-building optimization.

## Figures and Tables

**Table 1:** Summary of key information available for building neonatal PBPK models. The table describes parameters often considered in PBPK models, the age range where data is available, specifically the developmental pattern of the parameter early in life, strength of evidence (see footnote for definitions), and key references for data.

ADME Process	Parameter	Age Range Reported	Developmental Pattern Early in Life (Fetus 22 to 44 wk PMA)	Strength of Information in Neonates	Key References Containing Neonatal Data
Demographics					
	Age Distribution	Fetus to adult	Uniform distribution	3	Health survey for England, NHANES, Health data for specific country
	Height/length	Fetus to adult	Increasing	3	Growth Charts for specific population
	Weight	Fetus to adult	Increasing	3	Growth Charts for specific population
Absorption					
	Small intestinal length/diameter	Fetus to adult	Increase	3	[19,20,21,22,23]
	Gastric pH	Fetus to adult	Decreasing/stable	3	[24,25,26,27,28]
	Gastric emptying	Fetus to adult	Stable	3	[29] meta-analysis
	Small intestine transit time	Neonate to adult	Stable	1	[30]
	Intestinal transporters				[31,32,33,34,35]
	Pgp	Fetus to adult	Stable	2	
	BCRP	Fetus to adult	Stable	2	
	MRP1	Fetus to adult	Stable	1	
	OATP2B1	Neonate to adult	Decrease	2	
	Intestinal enzymes CYP3A4	Fetus to adult	Stable/Increasing	1	[16]
Distribution					
	Tissue composition (Individual organs)	Fetus, neonate, and adult	Changing	1	[36,37,38]
	Water Composition				[39,40,41]
	Intracellular Water	Fetus to adult	Increasing	3	
	Extracellular Water	Fetus to adult	Decreasing	3	
	Fat	Fetus to adult	Increasing	3	[20,42,43,44,45,46]
	**Organ Volumes**				
	Liver Volume	Fetus to adult	Increasing	3	[11,13,17,47,48]
	Brain Volume	Fetus to adult	Increasing	3	[11,13,20,49,50]
	Kidney Volume	Fetus to adult	Increasing	3	[11,13,51,52]
	Fat-Free Mass Volume	Fetus to adult	Increasing	3	[39,43]
	Blood Volume	Fetus to adult	Increasing	3	[53,54,55,56]
	**Organ Blood Flows**				
	Cardiac Output	Fetus to adult	Increasing	3	[57,58,59,60,61]
	Liver Blood Flow	Neonate to adult	Increasing with cardiac output	1	[62]
	Brain Blood Flow	Neonate to adult	Variable as fraction of cardiac output	2	[63,64]
	Kidney Blood Flow	Neonate to adult	Increasing	2	[65,66]
	**Blood Proteins**				
	Albumin Concentration	Fetus to adult	Slowly increasing	3	[15,67,68,69,70]
	AGP Concentration	Fetus to adult	Increasing	3	[15,69,71]
	Hematocrit	Fetus to adult	Decreasing/Increasing	3	[72,73,74]
Metabolism (Liver)					
	**Hepatic Enzymes**				
	CYP1A2	Fetus to adult	Slowly increasing	2	[75,76,77,78,79]
	CYP2A6	Fetus to adult	Slowly Increasing	1	[76,79,80]
	CYP2B6	Fetus to adult	Slowly Increasing	1	[79,81,82]
	CYP2C9	Fetus to adult	Slowly Increasing	2	[76,79,83,84,85,86]
	CYP2C19	Fetus to adult	Slowly Increasing	1	[76,79,83,84,86]
	CYP2D6	Fetus to adult	Slowly Increasing	1	[76,79,86,87]
	CYP2E1	Fetus to adult	Slowly Increasing	1	[79,83,88,89]
	CYP3A4	Fetus to adult	Stable/Slowly increasing	2	[76,83,90,91]
	CYP3A5	Fetus to adult	Stable	3	[76,83,91,92,93,94,95]
	CYP3A7	Fetus to adult	Decreasing	3	[79,83,90,91,92,96,97,98,99]
	UGT1A1	Neonate to adult	Stable	2	[100,101,102,103,104,105,106,107]
	UGT1A3	Neonate to adult	Decreasing/Increasing	1	[100,104,108]
	UGT1A4	Neonate to adult	Increasing	1	[101,109]
	UGT1A6	Neonate to adult	Slowly Increasing		[100,101,103,104,105]
	UGT1A9	Neonate to adult	Stable	1	[100,101,104,107,110,111]
	UGT2B4	Fetus to adult	Stable	1	[100,107]
	UGT2B7		Stable/increasing		[100,101,107,112,113]
	UGT2B15	Neonate to adult	Stable	1	[101]
	CES1	Neonate to adult	Slowly Increasing	1	[114,115,116]
	CES2	Neonate to adult	Slowly Increasing	1	[114,115,116,117]
	FMO1	Fetus to adult	Decreasing	1	[96]
	**Hepatic Transporters**				
	P-gp	Fetus to adult	Stable/slowly increasing	1	[31,118,119]
	BCRP	Neonate to adult	Stable	1	[118]
	OATP1B1	Fetus to adult	Stable/slowly increasing	1	[31,118,119]
	OATP1B3	Neonate to adult	Increasing	1	[31,118]
	OCT1	Neonate to adult	Increasing	1	[118]
	**Other**				
	Microsomal protein	Neonate to adult	Stable	1	[120]
Excretion					
	Glomerular filtration rate	Preterm to adult	Increasing	3	[121,122,123,124,125,126,127]
	**Renal Transporters**				
	BCRP	Preterm to adult	Decreasing	1	[128]
	P-gp	Preterm to adult	Increasing	1	[128]
	MATE1/2	Preterm to adult	Stable	1	[128]
	MRP4	Preterm to adult	Stable	1	[128]
	OAT1	Preterm to adult	Slowly Increasing	1	[128]
	OAT3	Preterm to adult	Slowly Increasing	1	[128,129,130]
	OCT2	Preterm to adult	Slowly Increasing	1	[128]

ADME = absorption, distribution, metabolism, and excretion. PMA = postmenstrual age. Strength of information—0 = no data, 1 = weak, 2 references or less or too few data points for ontogeny, 2 = moderate, >2 to 5 references and enough data for rough ontogeny estimate with PMA, 3 = strong, >3 references and rich data for analysis with PMA.

## Data Availability

Not applicable.
